# MicroRNA-497-5p stimulates osteoblast differentiation through HMGA2-mediated JNK signaling pathway

**DOI:** 10.1186/s13018-020-02043-4

**Published:** 2020-11-10

**Authors:** Huiqing Zhao, Yexiang Yang, Yang Wang, Xiaolei Feng, Adi Deng, Zhaolan Ou, Biying Chen

**Affiliations:** 1 Department of Orthopaedics, The Third Affiliated Hospital of Sun Yat-Sen University, No, 2693, Kaichuang Road, Guangzhou, 510530 Guangdong People’s Republic of China; 2 Department of Joint and Trauma Surgery, The Third Affiliated Hospital of Sun Yat-Sen University, Guangzhou, 510630 Guangdong People’s Republic of China; 3 Department of Spine Surgery, The Third Affiliated Hospital of Sun Yat-Sen University, No, 600, Tianhe Road, Tianhe District, Guangzhou, 510630 Guangdong People’s Republic of China

**Keywords:** MicroRNA-497-5p, HMGA2, JNK pathway, Osteoporosis, Osteoblasts

## Abstract

**Background:**

Osteoporosis (OP) has the characteristics of the decline in bone mineral density and worsening of bone quality, contributing to a higher risk of fractures. Some microRNAs (miRNAs) have been validated as possible mediators of osteoblast differentiation. We herein aimed to clarify whether miR-497-5p regulates the differentiation of osteoblasts in MC3T3-E1 cells.

**Methods:**

The expression of miR-497-5p in OP patients and controls was measured by RT-qPCR, and its expression changes during osteoblast differentiation were determined as well. The effects of miR-497-5p on the differentiation of MC3T3-E1 cells were studied using MTT, ALR staining, and ARS staining. The target gene of miR-497-5p was predicted by TargetScan, and the effects of its target gene on differentiation and the pathway involved were investigated.

**Results:**

miR-497-5p expressed poorly in OP patients, and its expression was upregulated during MC3T3-E1 cell differentiation. Overexpression of miR-497-5p promoted mineralized nodule formation and the expression of RUNX2 and OCN. miR-497-5p targeted high mobility group AT-Hook 2 (HMGA2), while the upregulation of HMGA2 inhibited osteogenesis induced by miR-497-5p mimic. miR-497-5p significantly impaired the c-Jun NH2-terminal kinase (JNK) pathway, whereas HMGA2 activated this pathway. Activation of the JNK pathway inhibited the stimulative role of miR-497-5p mimic in osteogenesis.

**Conclusions:**

miR-497-5p inhibits the development of OP by promoting osteogenesis via targeting HMGA2.

## Background

Bone protects other organs of the body, and fragility fracture in older people causes substantial morbidity and mortality, and measures to prevent such fractures involve promoting skeletal strength and lowering fall risk [1]. Moreover, vitamin D and calcium supplementation have been recommended as baseline treatment options in every patient with osteoporosis (OP) [2]. In addition, understanding the relationship among age-related comorbidities, fracture risk, and competing mortality risk is of paramount importance for practitioners caring for the OP patients at an older age [3]. OP is induced by the loss of bone mass because of the imbalance between bone formation modulated by osteoblasts and bone absorption modulated by osteoclasts, and the former one exhibits various characteristics during differentiation, including increased alkaline phosphate (ALP) activity, followed by extracellular matrix synthesis contributing to mineralization [4].

MicroRNAs (miRNAs, non-protein-coding RNAs) bind to target mRNAs which are complementary with their sequences and post-transcriptionally regulate gene expression through translational activation or repression such as mRNA degradation. Using this pathway, miRNAs play important roles in homeostatic processes including cell proliferation, differentiation, and cell death [5]. miRNAs are vital post-transcriptional modulators of gene expression that govern the osteoblast-mediated bone formation, thus involving in OP, osteoarthritis, and other bone-related disorders [6–8]. For instance, advanced PCR arrays adopted by a previous research team found that circulating hsa-miR-122-5p and hsa-miR-4516 exhibited potential diagnostic potentials for OP [9]. miR-497-5p was revealed to be significantly downregulated after 24 h stimulation of human primary osteoarthritis chondrocytes with IL-1β, indicating its relevance in joint disease [10]. Also, miR-497~195 cluster drives angiogenesis and osteogenesis, representing as an attractive therapeutic target for age-related OP [11]. More recently, miR-497-5p was found to be significantly reduced in bone tissues of aging and ovariectomized mice and upregulated during osteogenic differentiation of hFOB1.19 and MC3T3-E1 cells [12]. However, the downstream biomolecules of miR-497-5p in OP remains largely unknown. High mobility group AT-Hook 2 (HMGA2) is a chromatin-binding protein, commonly expressed during embryogenesis, and is undetectable in the majority of adult tissues and linked to multiple types of cancer [13]. HMGA2 specifically binds to AT-rich DNA sequences with its AT-hook DNA-binding motifs and triggers DNA bending. Although HMGA2 has an important role in adipogenesis and tumor formation, its main function is to bind to chromosomes to ensure that human embryonic stem cells retain stem cell strength, thereby maintaining the durability and renewal capacity of stem cells [14]. Interestingly, osteoblast differentiation was found to be induced by miR-33-5p, partially depending on HMGA2 [15]. Using TargetScan (http://www.targetscan.org/), we obtained the binding sites between miR-497-5p and HMGA2. As a consequence, we speculated that miR-497-5p participated in the pathology of OP by directly interacting with HMGA2 in osteoblasts. The significance of c-Jun N-terminal kinase (JNK) has been validated in cell cycle regulation, apoptosis, and cellular stress, and it has been also highlighted to participate in osteogenic differentiation of mesenchymal stem cells [16]. More specifically, miR-122 elicited inhibitory effects on osteoblast proliferation through the JNK pathway [17]. The present study hypothesized that miR-497-5p enhances the osteoblast differentiation by regulating HMGA2 and the JNK signaling pathway. To validate this hypothesis, the underlying mechanisms in MC3T3-E1 cells were investigated.

## Materials and methods

### Clinical samples

From January 2018 to March 2019, 15 patients who underwent hip replacement in The Third Affiliated Hospital of Sun Yat-Sen University for osteoporotic fractures were enrolled. Fifteen patients without OP who underwent traumatic arthroplasty were recruited as controls. All participants had no other metabolic or endocrine diseases. Tissues were immediately frozen at − 80 °C for subsequent experiments. The use of human samples was permitted by the Ethical Committee of The Third Affiliated Hospital of Sun Yat-Sen University, and informed consent was obtained from each participant.

### Cell culture, in vitro differentiation, and transfection

MC3T3-E1 cells (sub-clone 14, Cell Bank of Shanghai Institute of Cells, Chinese Academy of Science, Shanghai, China) were grown in α-Minimal Essential Medium (Gibco, Carlsbad, CA, USA) supplemented with 10% fetal bovine serum (Hyclone, Marlborough, MA, USA) and 1% penicillin and streptomycin (Thermo Fisher Scientific Inc., Waltham, MA, USA). The cells were kept at 37 °C under 5% CO_2_, and the culture medium was renewed every 2 days.

MC3T3-E1 cells were grown in a culture medium supplemented with 100 nM dexamethasone, 10 mM β-glycerol phosphate, and 50 μg/mL ascorbic acid for osteoblast differentiation. miR-497-5p mimic (which was synthesized chemically to enhance the function of endogenous miRNAs) and overexpression (oe)-HMGA2 or their respective controls (NC mimic or oe-NC) were purchased from GenePharma Ltd. Company (Shanghai, China). All transfection was performed using oligonucleotides and plasmids with Lipofectamine™ 2000 reagent (Invitrogen) in accordance with the manufacturer’s protocol. A JNK pathway-specific agonist azaspiracid-1 (AZA-1) was from Santa Cruz Biotechnology Inc. (10 nM, Santa Cruz, CA, USA, cargo number: sc-202482, CAS: 214899-21-5). Dimethylsulfoxide (DMSO) serves as a control (cargo number: sc-202581, CAS: 67-68-5, Santa Cruz Biotechnology Inc).

### Reverse transcription quantitative (RT-q) PCR

The extraction of total RNA was conducted using TRIzol reagents (Invitrogen). Complementary DNA (cDNA) was synthesized for mRNA using the PrimeScript RT kit (Takara Holdings Inc., Kyoto, Japan). Reverse transcription was conducted for miRNA using the PrimeScript miRNA cDNA Synthesis Kit (Takara). SYBR Premix Ex Taq I was employed for RT-qPCR. Relative expression of mRNA or miRNA was evaluated by the 2^−ΔΔCt^ method and normalized to glyceraldehyde-3-phosphate dehydrogenase (GAPDH) or 5S, respectively. The primers were as follows: miR-497-5p (human), forward 5′-CTCCCCCACCCTCGCTCTAA-3′ and reverse 5′-ACACTGTGGTTTGTACGGCA-3′; miR-497-5p (mouse), forward 5′-GCAGCACACTGTGGTTTG-3′ and reverse 5′-GAACATGTCTGCGTATCTC-3′; HMGA2 (human), forward 5′-GAAGCCACTGGAGAAAAACGGC-3′ and reverse 5′-GGCAGACTCTTGTGAGGATGTC-3′; HMGA2 (mouse), forward 5′-AGAGGAAGACCCAAAGGCAGCA-3′ and reverse 5′-GAGCAGGCTTCTTCTGAACGAC-3; Bax (mouse), forward 5′-AGGATGCGTCCACCAAGAAGCT-3′ and reverse 5′-TCCGTGTCCACGTCAGCAATCA-3′; BCl-2 (mouse), forward 5′-CCTGTGGATGACTGAGTACCTG-3′ and reverse 5′-AGCCAGGAGAAATCAAACAGAGG-3′; OCN (Bglap, mouse), forward 5′-GCAATAAGGTAGTGAACAGACTCC-3′ and reverse 5′-CCATAGATGCGTTTGTAGGCGG-3′; RUNX2 (mouse), forward 5′-CCTGAACTCTGCACCAAGTCCT-3′ and reverse 5′-TCATCTGGCTCAGATAGGAGGG-3′; GAPDH (human), forward 5′-GTCTCCTCTGACTTCAACAGCG-3′ and reverse 5′-ACCACCCTGTTGCTGTAGCCAA-3′; GAPDH (mouse), forward 5′-CATCACTGCCACCCAGAAGACTG-3′ and reverse 5′-ATGCCAGTGAGCTTCCCGTTCAG-3′; and 5S (human or mouse), forward 5′-CTCGCTTCGGCAGCACAT-3′ and reverse 5′-TTTGCGTGTCATCCTTGCG-3′.

### 3-(4,5-Dimethylthiazol-2-yl)-2,5-diphenyltetrazolium bromide (MTT) assay

An MTT cell proliferation and cytotoxicity assay kit was utilized to detect cell viability. Cells were added to 96-well plates at 2000 cells each well, and 10-μL MTT (5 mg/mL) solution was added to each well. After a 4-h incubation, 100 μL formazan lysate was supplemented to each well and incubated at 37 °C for about 3–4 h. The optical density (OD) value at 570 nm was measured on a microplate reader.

### Cell apoptosis by flow cytometry

An Annexin V-fluorescein isothiocyanate (FITC) apoptosis detection kit (Beyotime Biotechnology Co., Ltd., Shanghai, China) was applied to assess the apoptosis rate. Cells were resuspended in 195 μL Annexin V-FITC binding solution. The cells were mixed with 5 μL Annexin V-FITC and then stained with a 10-μL propidium iodide staining solution in the dark at 20–25 °C for 10–20 min. After an ice bath, the cells were loaded onto a flow cytometer.

### ALP staining

After a 7-day culture in osteogenic medium, ALP staining was carried out. Cells in six-well plates were fixed with 4% paraformaldehyde for 15 min. A BCIP/NBT Alp Color Development kit (Beyotime, Shanghai, China) was used for a 30-min staining at room temperature in the dark. Images were obtained by a digital camera.

### Alizarin red S (ARS) staining

MC3T3-E1 cells cultured in osteogenic medium for 21 days were fixed with ice-cold 70% ethanol for 40 min at 4 °C and stained in 1% ARS (Sigma, St. Louis, MO, USA) for 15 min at room temperature. The stained cells were then imaged by a digital camera.

### Dual-luciferase reporter genes constructs and assays

The binding sites between miR-497-5p and HMGA2 were predicted from TargetScan (http://www.targetscan.org/). The HMGA2 3′-untranslated region (3′UTR) with binding sites was amplified and cloned into pGL3 vectors (Promega) to obtain wild-type (WT) constructs. The binding sites were mutated to obtain HMGA2 mutant-type (MT) constructs. The above vectors were co-transfected into 293T cells (ATCC, Manassas, VA, USA) with the miR-497-5p mimic and its control, respectively. At 48 h post-transfection, luciferase activity was tested by the luciferase reporter assay system (Promega, Madison, WI, USA).

### RNA immunoprecipitation (RIP)

A RIP lysis buffer kit (Millipore Corp, Billerica, MA, USA) was utilized for RIP experiments. In brief, MC3T3-E1 cells were lysed in RIP lysis buffer and incubated with anti-AGO2 (Millipore Corp) and anti-IgG (Millipore Corp)-coupled A/G agarose particles. The precipitated RNA was isolated using TRIzol reagents, and gene expression was determined using RT-qPCR.

### Western blot

Radio-immunoprecipitation assay lysis buffer (Solarbio Science & Technology Co., Ltd., Beijing, China) with a proteinase inhibitor was used to extract the total proteins in cells. Next, a bicinchoninic acid assay protein assay kit (Thermo Fisher Scientific) was adopted for protein quantification. Lysates were electrophoresed on 10% sodium dodecyl sulfate-polyacrylamide gel electrophoresis and transferred to polyvinylidene difluoride membranes (Millipore) with 5% skim milk. The blots were probed with primary antibodies against HMGA2 (1:1000, #8179, Cell Signaling Technologies (CST), Beverly, MA, USA), JNK (1:1000, ab179461, Abcam, Cambridge, MA, USA), p-JNK (phospho T183 + T183 + T221, 1:5000, ab124956, Abcam), or GAPDH (1:1000, #5174, Cell Signaling Technologies) at 4 °C overnight and then probed with horseradish peroxidase-conjugated secondary goat anti-rabbit antibody IgG (1:10,000, ab205718, Abcam) at room temperature for a period of 2 h. Finally, the immunoblots were subjected to enhanced chemiluminescence reagent (Millipore).

### Statistical analysis

Calculations were performed with the SPSS 22.0 software (IBM Corp., Armonk, NY, USA). The data are expressed as the mean ± standard deviation (SD) of at least three independent experiments. Data between the two groups were compared by the unpaired *t* test and data among more than two groups by the two-way analysis of variance (ANOVA), followed by Tukey’s multiple comparison test. *p* < 0.05 was considered to be reflective of a statistically significant difference.

## Results

### miR-497-5p is increased during osteogenic differentiation

**Fig. 1:**
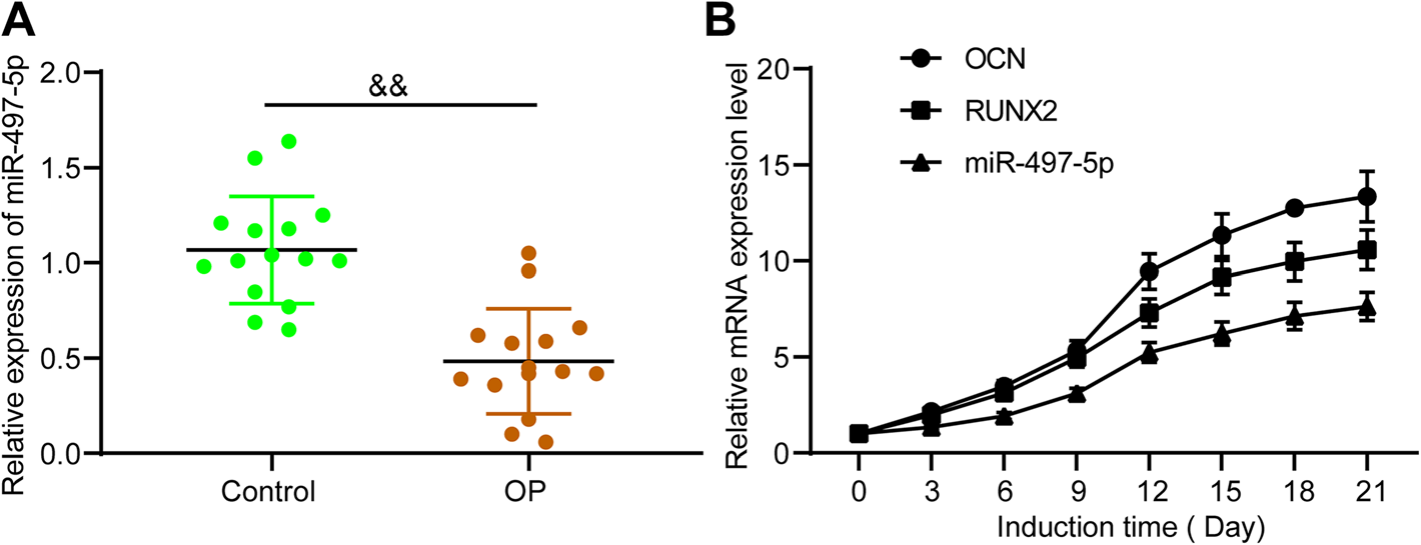
miR-497-5p expression during osteogenic differentiation. **a** RT-qPCR measurement of miR-497-5p expression in bone tissues of OP patients and controls. **b** RT-qPCR measurement of OCN, RUNX2, and miR-497-5p expression during osteogenic differentiation. Data are presented as the mean ± SD of three replicates each. The unpaired *t* test was applied for the comparison between the two groups. ^&&^*p* < 0.01 vs. controls

According to a previous report [11], miR-497-5p was believed to be a potential therapeutic target for OP, but the mechanism involved has not been studied. miR-497-5p expression was found to be significantly reduced in bone tissues of OP relative to controls, as revealed by RT-qPCR (Fig. 1a). MC3T3-E1 cells were cultured for 21 days; RT-qPCR was conducted to assess the expression of osteogenic markers and miR-497-5p. It was noted that the expression of these markers was increased gradually with the development of osteogenic culture (Fig. 1b).

### Overexpression of miR-497-5p promotes osteogenic differentiation

**Fig. 2:**
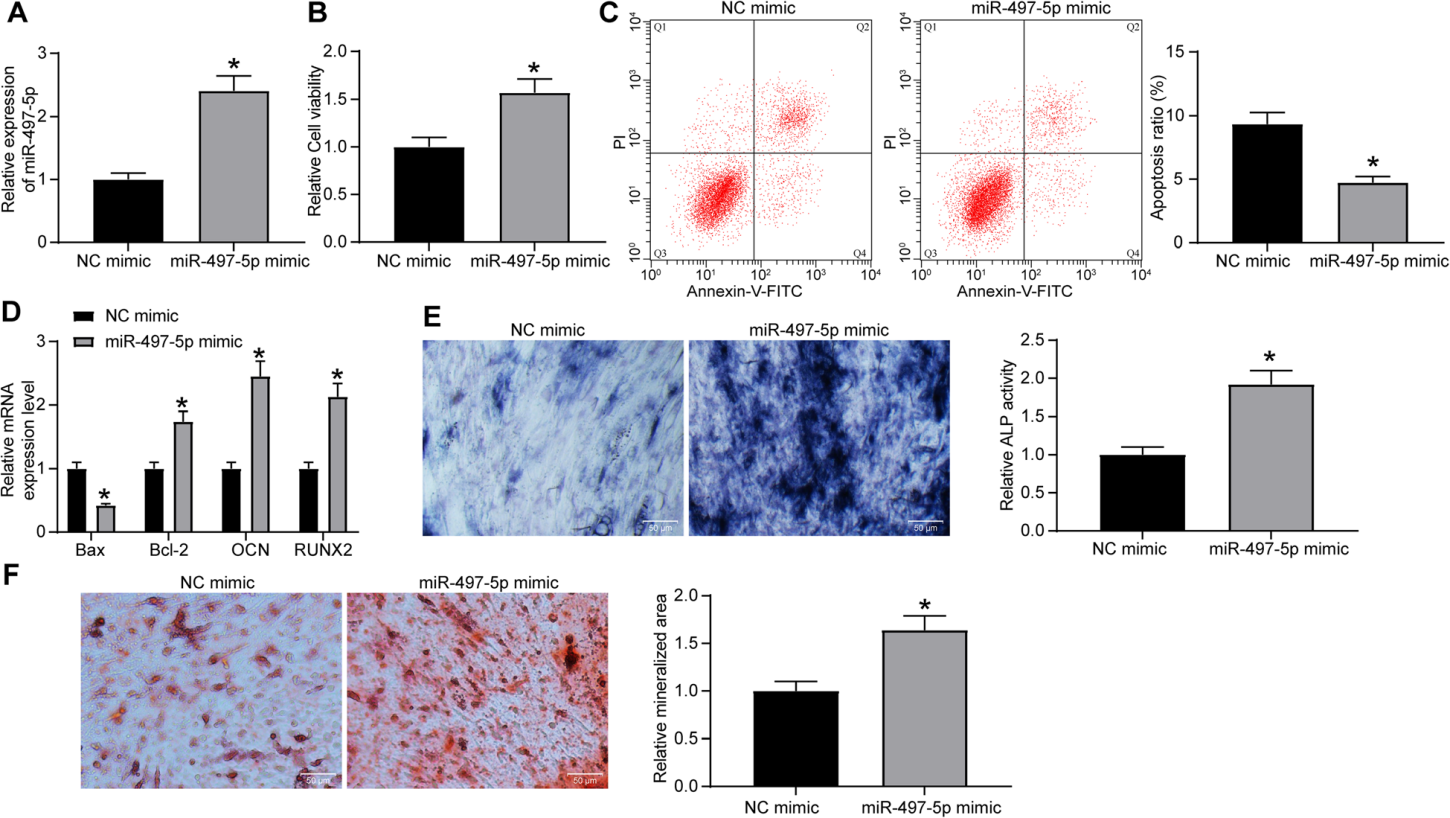
miR-497-5p promotes the differentiation of MC3T3-E1 cells. miR-497-5p mimic or NC mimic was transfected into MC3T3-E1 cells. **a** RT-qPCR measurement of miR-497-5p expression in MC3T3-E1 cells after transfection. **b** MTT assessment of MC3T3-E1 cell viability. **c** Flow cytometric analysis for cell apoptosis. **d** RT-qPCR measurement of Bax, Bcl-2, OCN, and RUNX2 expression. **e** ALP staining for ALP activity. **f** ARS staining for mineralization. Data are presented as the mean ± SD of three replicates each. The unpaired *t* test was used for the comparison between the two groups (**a**–**c**, **e**, **f**); the two-way ANOVA was applied for the comparison among multiple groups (**d**). *p < 0.05 vs. MC3T3-E1 cells transfected with NC mimic

miR-497-5p mimic and NC mimic were transfected into MC3T3-E1 cells, and RT-qPCR was used for effective transfection (Fig. 2a). The viability of cells was measured by MTT, and it was observed that miR-497-5p mimic significantly promoted the viability of cells (Fig. 2b), while flow cytometry found that miR-497-5p mimic also suppressed apoptosis (Fig. 2c). The expression of apoptotic factors Bax and Bcl-2 and osteogenic differentiation markers OCN and RUNX2 was measured by RT-qPCR at day 7 of osteogenic culture. miR-497-5p mimic notably enhanced the Bcl-2, OCN, and RUNX2 expression, while inhibited Bax expression (Fig. 2d). Meanwhile, ALP staining indicated that miR-497-5p mimic remarkably increased the ALP activity (Fig. 2e). On the 21st day of osteogenic culture, miR-497-5p was found to significantly promote mineralized nodule formation (Fig. 2f) by ARS staining.

### miR-497-5p targets HMGA2

**Fig. 3:**
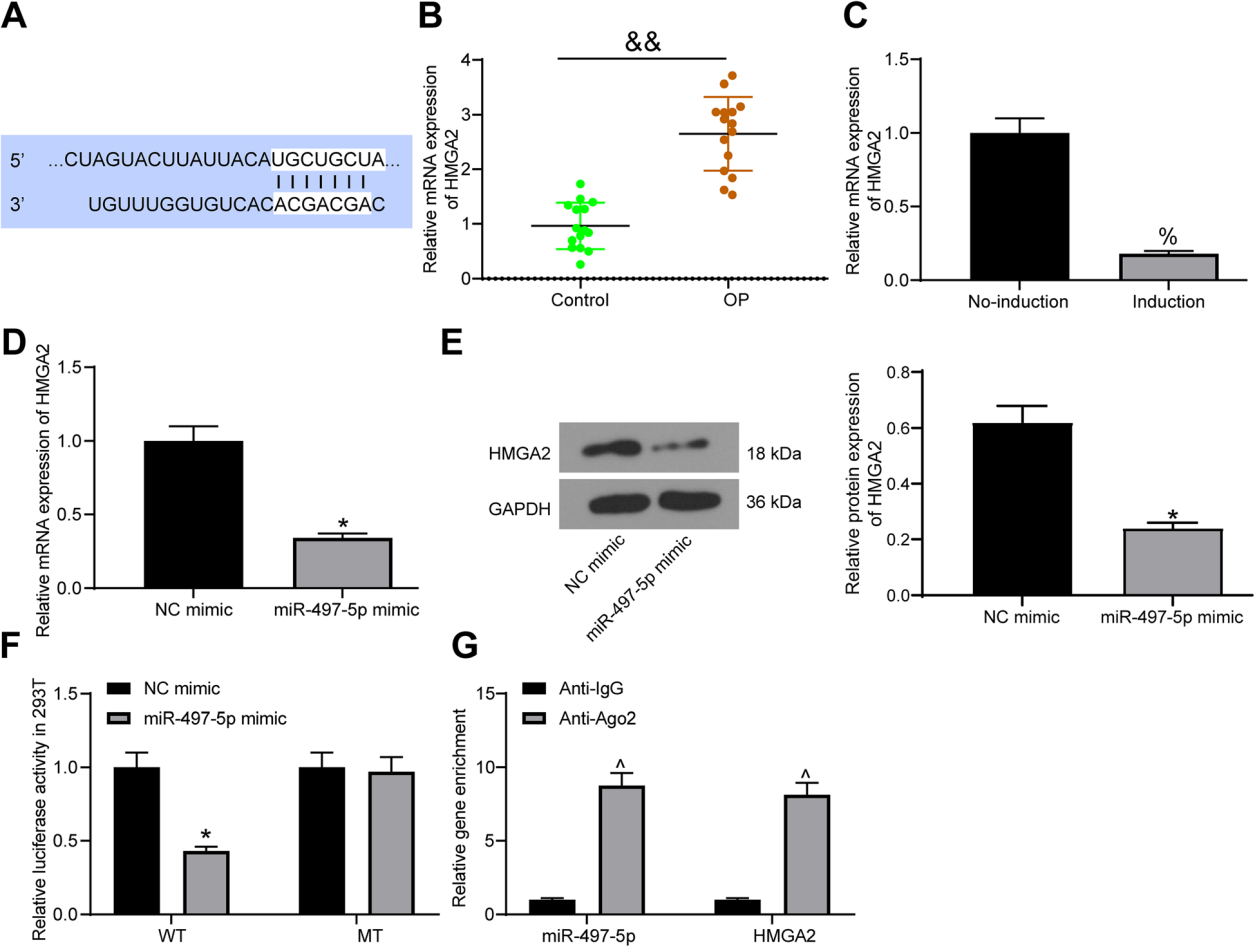
HMGA2 is verified to be a target gene of miR-497-5p. **a** Binding regions between HMGA2 3′UTR and miR-497-5p sequence. **b** RT-qPCR measurement of HMGA2 mRNA expression in bone tissues of OP patients and controls. **c** RT-qPCR measurement of HMGA2 mRNA expression during osteogenic differentiation. **d** RT-qPCR measurement of HMGA2 mRNA expression in MC3T3-E1 cells after transfection. **e** Western blot measurement of HMGA2 protein expression in MC3T3-E1 cells after transfection. **f** Luciferase activity of the HMGA2-WT and HMGA2-MT after transfection. **g** RIP validation of the relationship between miR-497-5p and HMGA2. Data are presented as the mean ± SD of three replicates each. The unpaired *t* test was used for the comparison between the two groups (**b**–**e**); the two-way ANOVA was applied for the comparison among multiple groups (**f**, **g**). ^&&^*p* < 0.01 vs. controls; ^%^*p* < 0.05 vs. cells before induction; *p < 0.05 vs. MC3T3-E1 cells transfected with NC mimic; ^^^*p* < 0.05 vs. cells treated with Anti-IgG

A bioinformatics website TargetScan predicted the targeting relationship between miR-497-5p and HMGA2 (Fig. 3a). HMGA2 was previously thought to inhibit osteogenic differentiation of stem cells [18, 19], so we speculated that it was a target of miR-497-5p to play a part in OP. HMGA2 was observed to be overexpressed (Fig. 3b) in bone tissues of OP patients relative to controls. At the same time, HMGA2 was downregulated (Fig. 3c) after osteogenic differentiation. miR-497-5p mimic significantly inhibited HMGA2 mRNA and protein expression in MC3T3-E1 cells (Fig. 3d, e). Luciferase reporter experiments in 293T cells showed that miR-497-5p mimic significantly decreased luciferase activity in HMGA2-WT, but had no significant effect on HMGA2-MT (Fig. 3f). Meanwhile, RIP experiments displayed that anti-Ago2 significantly enriched miR-497-5p and HMGA2 (Fig. 3 g) compared to anti-IgG. We thus established the targeting relationship between miR-497-5 and HMGA2.

### Overexpression of HMGA2 attenuates the effects of miR-497-5p mimic and activates the JNK pathway

**Fig. 4:**
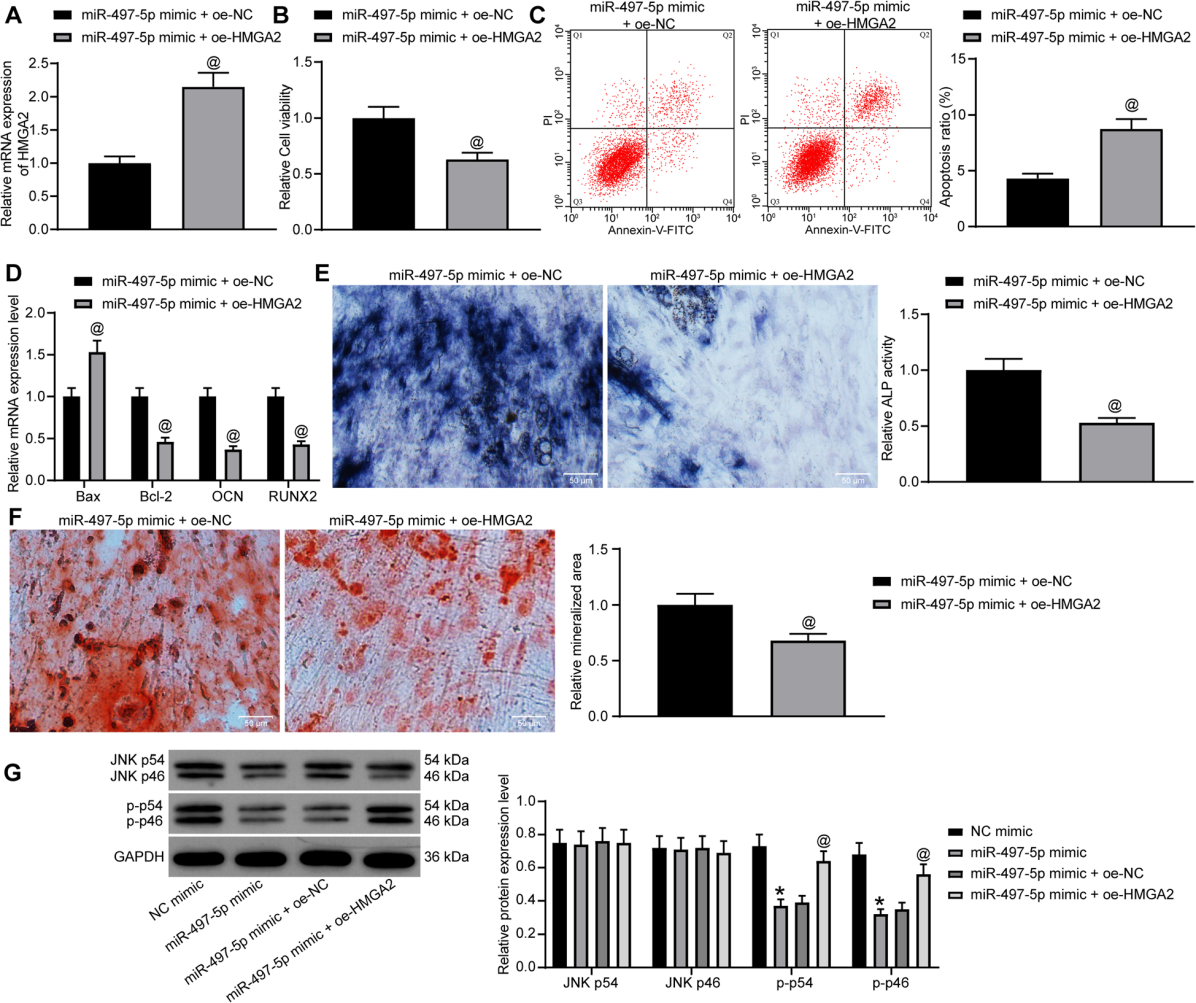
HMGA2 inhibits the differentiation of MC3T3-E1 cells. miR-497-5p mimic + oe-HMGA2 or miR-497-5p mimic + oe-NC was transfected into MC3T3-E1 cells. **a** RT-qPCR measurement of HMGA2 expression in MC3T3-E1 cells after co-transfection. **b** MTT assessment of MC3T3-E1 cell viability. **c** Flow cytometric analysis for cell apoptosis. **d** RT-qPCR measurement of Bax, Bcl-2, OCN, and RUNX2 expression. **e** ALP staining for ALP activity. **f** ARS staining for mineralization. **g** Western blot detection of activation of the JNK pathway. Data are presented as the mean ± SD of three replicates each. The unpaired *t* test was used for the comparison between the two groups (**a**–**c**, **e**, **f**); the two-way ANOVA was applied for the comparison among multiple groups (**d**, **g**). *p < 0.05 vs. MC3T3-E1 cells transfected with NC mimic; ^@^*p* < 0.05 vs. MC3T3-E1 cells transfected with miR-497-5p mimic + oe-NC

oe-HMGA2 was introduced into cells transfected with miR-497-5p mimic to generate cells overexpressing miR-497-5p and HMGA2 simultaneously, and RT-qPCR confirmed that the transfection was effective (Fig. 4a). MTT revealed that oe-HMGA2 significantly inhibited cell viability (Fig. 4b). Flow cytometry found that oe-HMGA2 attenuated the repressive role of miR-497-5p mimic on apoptosis (Fig. 4c). The expression of pro-apoptotic factors Bax and Bcl-2 and osteogenic differentiation markers OCN and RUNX2 in cells after co-transfection was measured by RT-qPCR at day 7 of osteogenic culture. oe-HMGA2 was found to lead to increased Bax expression, while decreased expression of Bcl-2, OCN, and RUNX2 (Fig. 4d). ALP staining found that oe-HMGA2 resulted in decreased activity ALP (Fig. 4e). By day 21, ARS staining revealed that oe-HMGA2 reduced mineralized nodule formation (Fig. 4f). Western blot displayed that miR-497-5p mimic significantly inhibited JNK phosphorylation, whereas this inhibition was significantly reversed by oe-HMGA2 (Fig. 4 g).

### The activation of the JNK pathway abrogates miR-497-5p mimic promotion on osteogenesis

**Fig. 5:**
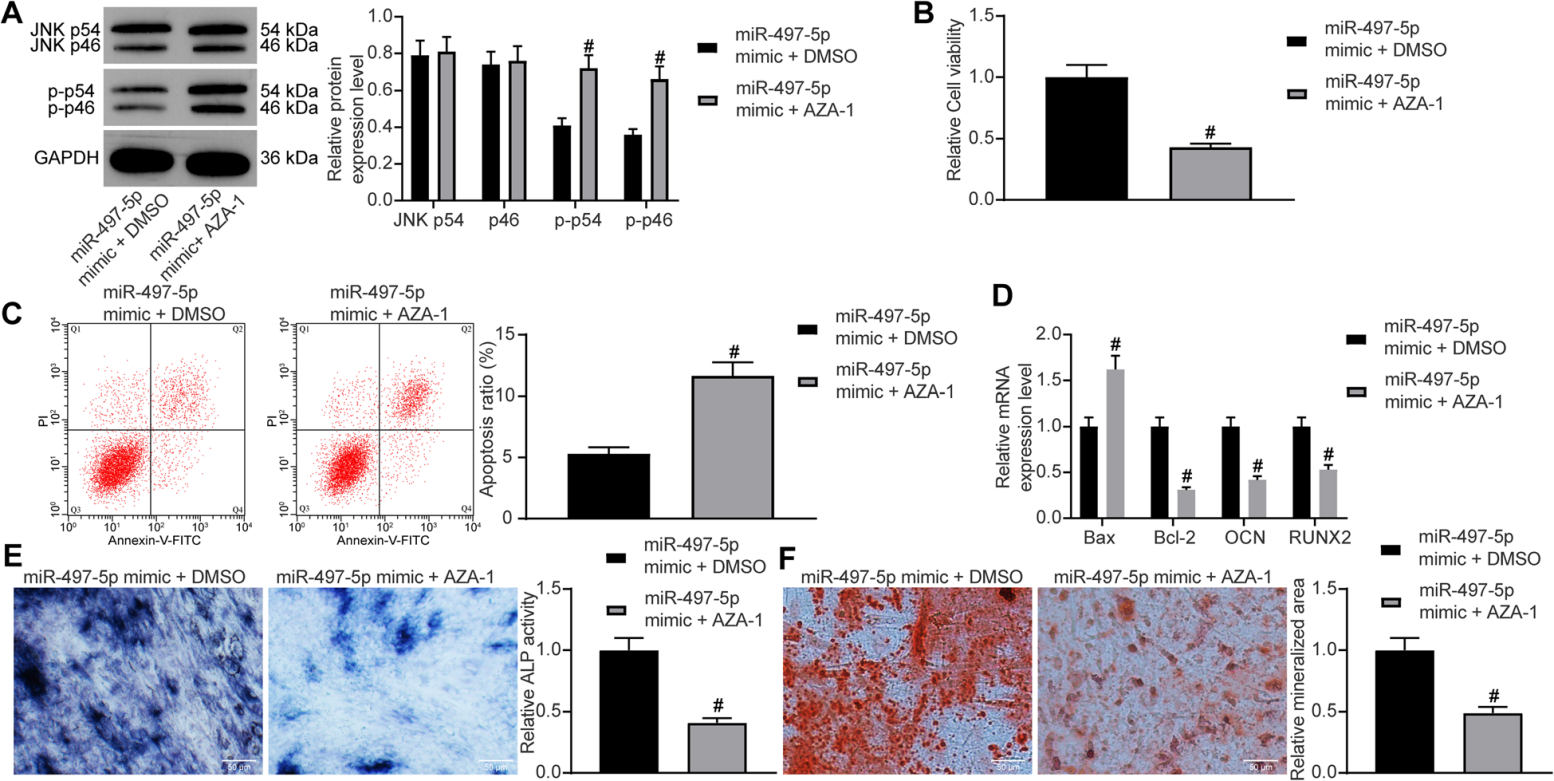
The potentiation of the JNK pathway ameliorates the stimulative role of miR-497-5p mimic on differentiation. miR-497-5p mimic + AZA-1 or miR-497-5p mimic + DMSO was delivered into MC3T3-E1 cells. **a** JNK pathway activity in transfected MC3T3-E1 cells. **b** MTT assessment of MC3T3-E1 cell viability. **c** Flow cytometric analysis for cell apoptosis. **d** RT-qPCR measurement of Bax, Bcl-2, OCN, and RUNX2 expression. **e** ALP staining for ALP activity. **f** ARS staining for mineralization. Data are presented as the mean ± SD of three replicates each. The unpaired *t* test was used for the comparison between the two groups (**b**, **c**, **e**, **f**); the two-way ANOVA was applied for the comparison among multiple groups (**a**, **d**). ^#^*p* < 0.05 vs. MC3T3-E1 cells administrated with miR-497-5p mimic + DMSO

A JNK pathway-specific agonist AZA-1 was delivered into MC3T3-E1 overexpressing miR-497-5p with DMSO as a control. The phosphorylation of the JNK pathway was significantly promoted, as revealed by Western blot (Fig. 5a). Meanwhile, AZA-1 was found to inhibit cell proliferation and promote apoptosis by CCK-8 and flow cytometry (Fig. 5b, c). The expression of apoptotic factors Bax and Bcl-2 and osteogenic differentiation markers OCN and RUNX2 in cells after delivery of AZA-1 and miR-497-5p mimic was measured by RT-qPCR at day 7 of osteogenic culture. The expression of Bax was significantly increased after the activation of the JNK pathway, while the expression of Bcl-2, OCN, and RUNX2 was significantly decreased (Fig. 5d). Meanwhile, ALP staining exhibited a decrease in ALP activity following miR-497-5p mimic + AZA-1 administration (Fig. 5e). On the 21st day of the culture, a decrease in deposition of calcium (Fig. 5f) was found by ARS staining, which illustrated that potentiation of the JNK pathway significantly attenuated the osteogenesis induced by the miR-497-5p mimic.

## Discussion

Older people with hip fracture benefitted more from comprehensive care involving interdisciplinary care, depression management, and fall prevention [20]. MC3T3-E1 is a popular osteoblast cell line with a pre-osteoblastic phenotype, and its sub-clone 14 has been shown to mineralize the collagenous extracellular matrix [21], which makes it an ideal tool for in vitro investigations regarding bone remodeling and formation [22, 23]. The results of the current investigation displayed that miR-497-5p was remarkably upregulated in the process of osteogenic differentiation in vitro and that HMGA2 might be a direct target of miR-497-5p. miR-497-5p upregulation enhanced ALP activity and the expression patterns of osteoblast markers, including OCN and RUNX2. In addition, our observations here demonstrated that miR-497-5p upregulation inhibited MC3T3-E1 cell apoptosis. miR-497-5p disrupted the JNK pathway by binding to HMGA2.

The essential roles of numerous miRNAs played in bone development and homeostasis have been underscored, particularly in osteoblast differentiation [24]. miR-497-5p, significantly decreased in OP patients, was found to be elevated during osteogenic differentiation in MC3T3-E1 cells. The miR-497~195 cluster, reported by Grünhagen et al. to be related to osteoblast differentiation, encompasses mmu-miR-497 coding for miR-497-5p, which shares the highest similarity to miR-15a and 15b, and mmu-miR-195a encoding miR-195-5p, which is identical to miR-16 [25]. In line with our study, miR-497-5p was downregulated in osteoarthritis cartilage, while miR-497-5p overexpression attenuated cartilage matrix degradation stimulated by IL-1β in chondrocytes [26]. ALP, which is expressed by osteoblasts, is an important marker of bone mineralization, and Alizarin red staining is frequently applied to assess mineralization [27]. The results of the present study illustrated that osteogenesis in MC3T3-E1 cells treated with miR-497-5p mimic was more robust relative to those treated with NC mimic, as indicated by higher ALP activities and stronger formation of mineralized nodules.

In addition, HMGA2, overexpressed in OP patients and downregulated following osteogenesis, was revealed as a putative target of miR-497-5p. Consistently, Kalomoiris et al. found that within 9 days of culturing, the expression of HMGA2 quickly decreases during the early expansion of mesenchymal stem cells [13]. Moreover, let-7, another miRNA, positively modulates osteogenic differentiation by repressing HMGA2 [18]. Also, the impaired osteogenic differentiation of inflamed dental pulp stem cells was linked to the promoted expression of HMGA2 and the extent of PI3K and Akt phosphorylation [28]. In the current work, upregulation of HMGA2 contributed to the enhanced Bax expression; lowered Bcl-2, OCN, and RUNX2 expression along with curtailed ALP activity; and mineralized nodules in the presence of miR-497-5p mimic, indicating that HMGA2 overexpression reversed the promotive role of miR-497-5p in osteogenesis. In line with our findings, HMGA2 weakened the osteogenic differentiation of bone marrow-derived mesenchymal stem cells stimulated by miR-664a-5p [19]. More specifically, the reduction of HMGA2 expression alone promoted the osteogenic differentiation and calcium deposition in mesenchymal stem cells [29]. Consequently, we may draw a conclusion that the stimulative role of miR-497-5p played in MC3T3-E1 cells was reached by the interaction with HMGA2. Our observations proposed that the JNK signaling potentiation using an agonist AZA-1 antagonized the role of miR-497-5p mimic in MC3T3-E1 cells, further supporting the involvement of the JNK signaling in osteoblast differentiation.

Our study also showed that miR-497-5p impaired the JNK signaling potentiation by lowering the extents of JNK phosphorylation, which was also reversed by HMGA2 upregulation. Recently, the JNK signaling pathway inhibitor was revealed to enhance osteoblast differentiation [17]. Pre-treatment with MAPK inhibitors reduced the protein expression of Bax promoted by IL-1α in the MC3T3-E1 cells, suggesting the significance of JNK and the p38 MAPK signaling in modulating IL-1α-induced apoptosis and osteoblast differentiation of MC3T3-E1 cells [30]. In addition, phosphorylation of JNK leads to an increase in apoptosis rate in cells under different conditions, such as ischemia-reperfusion [31, 32], cancers [33, 34], and liver injury [35]. Moreover, miR-214 inhibitor significantly decreased the expression of ALP, OCN, and RUNX2, as well as ALP activity in MC3T3-E1 cells, which was enhanced by additional treatment with SP600125, a JNK inhibitor [36]. These existing reports validated the inhibitory effects of the JNK signaling in osteoblast differentiation. The negative correlation between miR-497 and the JNK signaling has also been highlighted in non-small cell lung cancer [37].

## Conclusion

In summary, we showed that miR-497-5p enhanced osteogenic differentiation by repressing HMGA2 and impairing the JNK signaling. However, as this study is based on the MC3T3-E1 cell line, we remain uncertain about the effects of miR-497-5p in vivo, an issue that must be addressed before ever proceeding to translational studies.

## Data Availability

All the data generated or analyzed during this study are included in this published article.

## Abbreviations

ALP: Alkaline phosphatase
ARS: Alizarin red S
cDNA: Complementary DNA
FITC: Fluorescein isothiocyanate
GAPDH: Glyceraldehyde-3-phosphate dehydrogenase
HMGA2: High mobility group AT-Hook 2
JNK: c-Jun NH2-terminal kinase
miRNAs: MicroRNAs
MT: Mutant
MTT: 3-(4,5-Dimethylthiazol-2-yl)-2,5-diphenyltetrazolium bromide
OD: Optical density
oe: Overexpression
OP: Osteoporosis
RIP: RNA immunoprecipitation
RT: Reverse transcription
SD: Standard deviation
WT: Wild-type
3′UTR: 3′-Untranslated region
